# A global gene evolution analysis on *Vibrionaceae *family using phylogenetic profile

**DOI:** 10.1186/1471-2105-8-S1-S23

**Published:** 2007-03-08

**Authors:** Nicola Vitulo, Alessandro Vezzi, Chiara Romualdi, Stefano Campanaro, Giorgio Valle

**Affiliations:** 1 CRIBI Biotechnology Centre, Department of Biology, University of Padova, Padova, Italy

## Abstract

**Background:**

*Vibrionaceae *represent a significant portion of the cultivable heterotrophic sea bacteria; they strongly affect nutrient cycling and some species are devastating pathogens.

In this work we propose an improved phylogenetic profile analysis on 14 *Vibrionaceae *genomes, to study the evolution of this family on the basis of gene content.

The phylogenetic profile is based on the observation that genes involved in the same process (e.g. metabolic pathway or structural complex) tend to be concurrently present or absent within different genomes. This allows the prediction of hypothetical functions on the basis of a shared phylogenetic profiles. Moreover this approach is useful to identify putative laterally transferred elements on the basis of their presence on distantly phylogenetically related bacteria.

**Results:**

*Vibrionaceae *ORFs were aligned against all the available bacterial proteomes. Phylogenetic profile is defined as an array of distances, based on aminoacid substitution matrixes, from single genes to all their orthologues. Final phylogenetic profiles, derived from non-redundant list of all ORFs, was defined as the median of all the profiles belonging to the cluster. The resulting phylogenetic profiles matrix contains gene clusters on the rows and organisms on the columns.

Cluster analysis identified groups of "core genes" with a widespread high similarity across all the organisms and several clusters that contain genes homologous only to a limited set of organisms. On each of these clusters, COG class enrichment has been calculated. The analysis reveals that clusters of core genes have the highest number of enriched classes, while the others are enriched just for few of them like DNA replication, recombination and repair.

**Conclusion:**

We found that mobile elements have heterogeneous profiles not only across the entire set of organisms, but also within *Vibrionaceae*; this confirms their great influence on bacteria evolution even inside the same family. Furthermore, several hypothetical proteins highly correlate with mobile elements profiles suggesting a possible horizontal transfer mechanism for the evolution of these genes. Finally, we suggested the putative role of some ORFs having an unknown function on the basis of their phylogenetic profile similarity to well characterized genes.

## Background

Over the past ten years, a great number of microbial genomes have been sequenced covering a wide representation of prokaryots as well as multiple strains of some species. The study of these genomes both by computational and experimental approaches has highly improved our understanding on physiology, phylogenetic relationship and pathogenicity of many organisms. Furthermore, it has provided new knowledge on microbial genome evolution, revealing a gene core shared by the great majority of bacteria, genes characteristic of particular groups and "novel" genes that possibly originated by lateral gene transfer from some unknown source.

Analysis performed on closely related genomes revealed that a substantial fraction of genes in any genome seem to be strain specific. These genes might sometime arise by gene duplication followed by a rapid divergence, or by lineage-specific loss of genes in one strain, resulting in a unique gene in other strains. However, there are several lines of evidence indicating that lateral gene transfer may be the main mechanism to acquire novel genes. Indeed, this could be one of the main forces driving bacterial adaptation and evolution. Phage DNA is thought to be one of the main vectors for lateral gene transfer among bacteria [1] and many virulence factors from bacterial pathogen are phage encoded [2]. For example, the genes for CT, the most important virulence factor of *V. cholerae*, are encoded in the genome of phage CTX*φ*, integrated in the bacterial chromosome 1.

Since lateral gene transfer plays a relevant role in bacterial evolution, the reconstruction of phylogeny is very complex and phylogenetic trees built by standard sequence analysis may not lead to a reliable picture of the evolutionary history. In fact, alternative trees can be obtained when different proteins are considered.

For many aspects the classification of bacteria on the basis of their global gene content may give a better description of their evolutionary history. This may be particularly important when bacteria of the same group are compared, since newly acquired genes could be relevant to confer peculiar features that allows the exploitation of different ecological niches.

In this study we propose a bioinformatic procedure to investigate bacterial genome evolution, taking into account the global gene content, as well as sequence similarity. We based our analysis on modified phylogenetic profiles [3]; however, we do not consider only the presence/absence of orthologue genes, but also their distance, based on a substitution matrix.

A phylogenetic profile is a non-sequence-homology-based method developed to infer a possible functional relationship between genes. It is based on the idea that proteins that are involved in the same metabolic pathway or structural complex are likely to evolve in a correlate fashion and during evolution appear phylogenetically linked, showing a tendency to be either preserved or eliminated as a whole. Therefore, genes showing similar phylogenetic profiles are likely to be functionally related. We extended the use of phylogenetic profiles to produce an evolutionary tree based on a hierarchical clusterization of organisms with similar phylogenetic profiles.

For this study we took the whole gene dataset of 320 prokaryotic genomes, however, we limited the analysis to the orthologous groups that are present in at least one of the 14 considered species of the *Vibrionaceae *family. These bacteria belong to the *Gammaproteobacteria *group and are highly abundant in aquatic environment, they strongly influence nutrient cycling and various species are also devastating pathogens. Since we focused our analysis on this particular group, the aim of this study is not the construction of a global evolutionary tree, but rather a *Vibrionaceae *perspective of bacterial diversity, based on phylogenetic profiles.

## Results and discussion

### Phylogenetic matrix

The analysis was performed on 14 bacteria belonging to the *Vibrionaceae *family (Table 1). The redundant list of *Vibrionaceae *ORFs was clustered to reduce the number of proteins to analyze and the phylogenetic profile for each cluster was calculated as described in the Method section.

Many authors proposed and successfully applied different measure methods to calculate the phylogenetic profile values.

Pellegrini *et al*. [3] firstly proposed a phylogenetic profile described as a string of bits, each bit representing the absence or presence of an homologous gene in a given genome. This method lacks a weighting procedure, giving the same weight (value 1) to all the sequences that are considered homologous given a similarity threshold. Enault and colleagues proposed an improved phylogenetic profile based on a normalized Blastp bit score [4]. This method, compared to the approach implemented by Pellegrini, allows weighting each point of the profile proportionally to the length and the quality of the alignment. Jingchun and colleagues optimized the phylogenetic profiles method by integrating phylogenetic relationships among reference organisms and sequence homology information, based on E-value score, to improve prediction accuracy [5].

The measure index *I *proposed in this work is similar to the others described above, taking into account both the quality and the length of the alignment using a substitution matrix. Moreover our approach considers also the total length of the sequences, penalizing good alignments occurring between ORFs having different lengths and taking into consideration that ORFs could differentiate mainly for the presence of functional domains.

The final phylogenetic profile for each cluster was defined as the median of all the profiles belonging to the cluster, named "meta-profile", which describes the profile of conserved ORFs belonging to an entire family.

### Hierarchical cluster analysis

**Figure 1:**
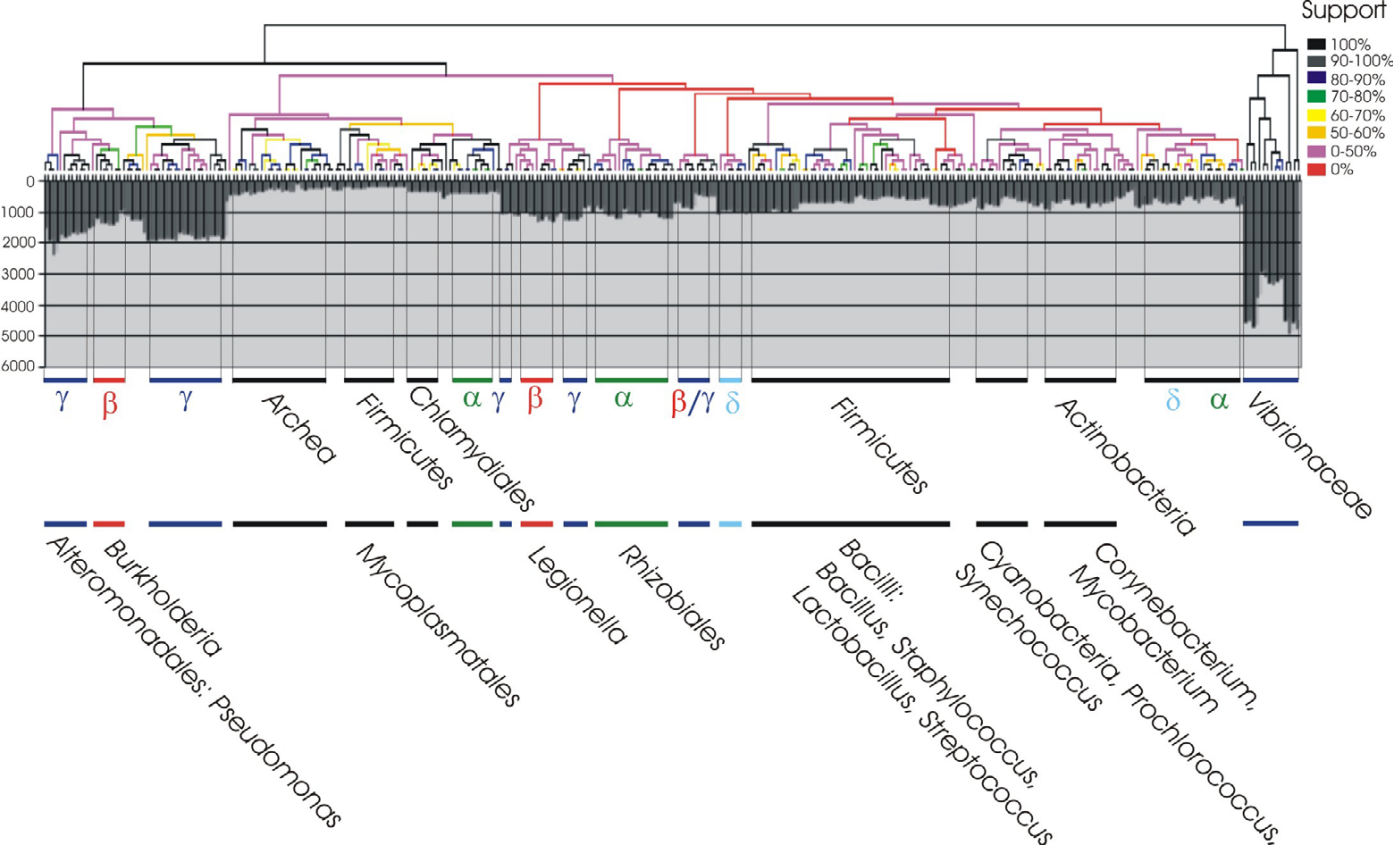
Hierarchical cluster analysis with bootstrap resampling method was performed on the complete set of organisms (columns of the phylogenetic profile matrix). The number of genes identified in each organism (with a similarity measure greater than zero) is reported as a gray histogram below the dendrogram. Organism taxonomies are highlighted with different colors: *γ *proteobacteria in blue, *β *proteobacteria in red, *α *proteobacteria in green, *δ *proteobacteria in light blue and others in black.

A hierarchical cluster analysis was performed on the entire phylogenetic profile matrix and it was calculated a statistical support based on bootstrap method for the nodes of the columns tree (Fig 1). The branch tree colors represent the bootstrap percentage support. This constitutes a phylogenetic tree based on gene content using *Vibrionaceae *ORFs as a reference. Genomes belonging to the same taxonomic group tend to cluster together and the *Vibrionaceae *species are closely related. As expected, according to the *Vibrionaceae *branch lengths it is evident that variability within this group is higher compared to the other groups. The dataset used for phylogenetic matrix calculation is indeed composed by *Vibrionaceae *ORFs. This implies that the similarity measures between these ORFs and the corresponding orthologues will be nearly zero in most of the other species and significantly higher in the *Vibrionaceae *family, increasing the variability into this group. Moreover the average percentage of clusters shared by the *Vibrionaceae *members is only 47.5% (average number of shared clusters divided by the total number of clusters) that again indicates a high variability inside this family. It is also interesting to note that organisms belonging to the same or closely related taxa split into different subgroups. This highlights the existence of a high variability among lineages, due to genetic and evolutionary processes such as lateral gene transfer, concerted evolution and gene duplication [6]. In terms of gene content, the organisms more related to the *Vibrionaceae *belong to the gamma and beta *proteobacteria*. In particular *Altermonadales*, *Enterobacteriales *and *Burkholderiales *are closely related to *Vibrionaceae*, and share the higher number of cluster of genes (average percentage of 20%). As expected, *Archea *are the most distant group sharing just 3.8% of clusters.

**Figure 2:**
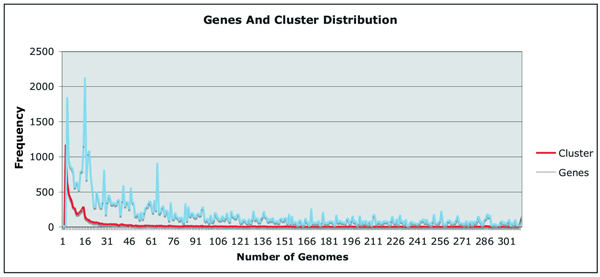
The blue line represents the number of genes, while the red line reports the number of gene clusters shared by an increasing number of genomes.

Clusters and genes distribution, as shown in Fig 2, reveals that the number of clusters and genes shared by the organisms decreases as the number of organisms considered increases. The analysis was performed considering for each cluster profile the number of organisms sharing the same numbers of clusters (and genes). The majority of gene cluster groups no more than 21 species on a total of 320. The highest blue spike corresponds to the higher number of genes shared by 105 groups of 14 organisms. Among these groups, as expected, *Vibrionaceae *are highly represented. Other species represented are *Colwellia psychrerythraea *34H and *Shewanella oneidensis*, that belong to the *Alteromonadales *family.

**Figure 3:**
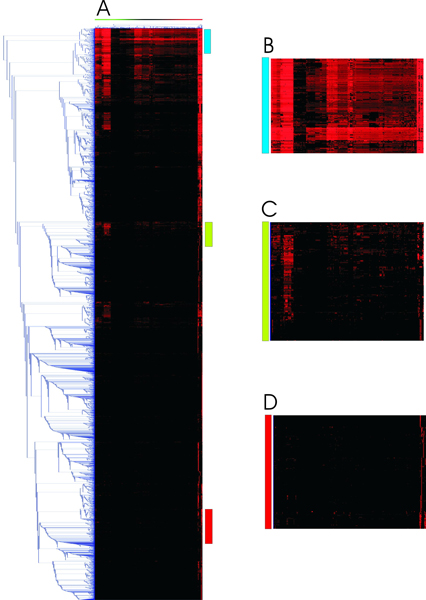
Two-way hierarchical cluster analysis of the entire phylogenetic profile matrix (panel A). Panel B: dendrogram selection zoom of highly conserved genes shared among all the organisms; panel C: genes conserved mostly among *gamma proteobacteria*; panel D: genes specific of *Vibrionaceae *family.

The cluster analysis performed on genes is shown in Fig. 3. From now on, to avoid confusing interpretation between clusters derived from the cluster analysis and cluster derived from the ORFs clustering we will use the term "gene" in place of cluster of ORFs.

The different gradient of color, from bright to dark red, represents decreasing similarity values. The cluster analysis allows the detection of three main groups of genes. The first one (Fig 3, panel B) contains the most conserved and established genes shared almost by all the organisms. These core genes can be defined as the set of all genes shared as orthologous by all members of an evolutionary coherent group. In our analysis we identify four clusters, for a total of 145 genes, shared by all the 320 organisms.

The ORFs belonging to these clusters are predicted to codify for the ATP binding subunit of ABC transporters (annotated as ABC-type polar amino acid transport system, ABC-type antimicrobial peptide transport system, ABC-type histidine transport system and ABC-type transport system involved in lysophospholipase L1 biosynthesis). This finding is surprising since this is the first report where these ORFs are assigned to the core genes. Anyway two different explanations can be traced. First, it is known that the ABC transporters represent an essential transport system in the prokaryotes and that their ATP binding subunits are apparently overrepresented compared to the other two subunits (ligand binding and permease subunit) in all genomes sequenced thus far [7]. Second, one organism, *Buchnera aphidicola*, presents these genes with a similarity just below the cut-off used for the analysis, but they have been considered since it is well known that in this mutualistic endosymbiont the accelerated evolution and AT bias affect all its genes, including the 16S rRNA [8,9].

The dataset used for the analysis includes genomes in draft quality (*Vibrio cholerae *0395, *Vibrio cholerae *MO10, *Vibrio cholerae *RC385, *Vibrio cholerae *V51, *Vibrio cholerae *V52, *Photobacterium profundum *3TCK, *Vibrio *MED222, *Vibrio splendidus*12B01). Wrong ORFs prediction or missing genes due to incomplete genome sequences can explain the low number of core genes identified. To avoid such problems we repeated the analysis excluding the draft genomes and thus considering 312 genomes. The results, reported in Table 2, show an increased number of the core genes and in particular ribosomal proteins and tRNA synthetase, as reported by Charlesbois and Doolittle [10]. This could be considered as a sort of "minimal genome" containing the group of genes that are necessary to maintain a free-living organism.

The low number of genes shared by all the organisms can be due to many factors. First of all we used the *Vibrionaceae *ORFs as a reference, limiting the number of genes we were able to identify. It was further demonstrated that the core gene size decreases as more genome sequences are analyzed [10].

Genes that are considered to belong to the core set when close organisms are compared, are classified as flexible genes when distantly related genomes are analyzed [6]. Finally, genes within core genomes might be transferred or replaced, introducing new versions of existing genes into genomes. Such transfers can replace even highly conserved genes by non-homologous counterparts but the advantages provided are difficult to explain. It is also to take into consideration that many symbiotic and parasitic bacteria undergo a reduction of their genomes, loosing many genes required by free-living cell.

The second group (Fig 3, panel C) represents genes shared mainly among *Vibrionaceae *and other gamma proteobacteria (as *Altermonadales*, *Burkholderiales *and *Enterobactidiales*).

Finally, the third group (Fig. 3, panel D) is composed by genes that are mainly specific to the *Vibrionaceae*.

### *k*-mean cluster analysis and cluster enrichment

**Figure 4:**
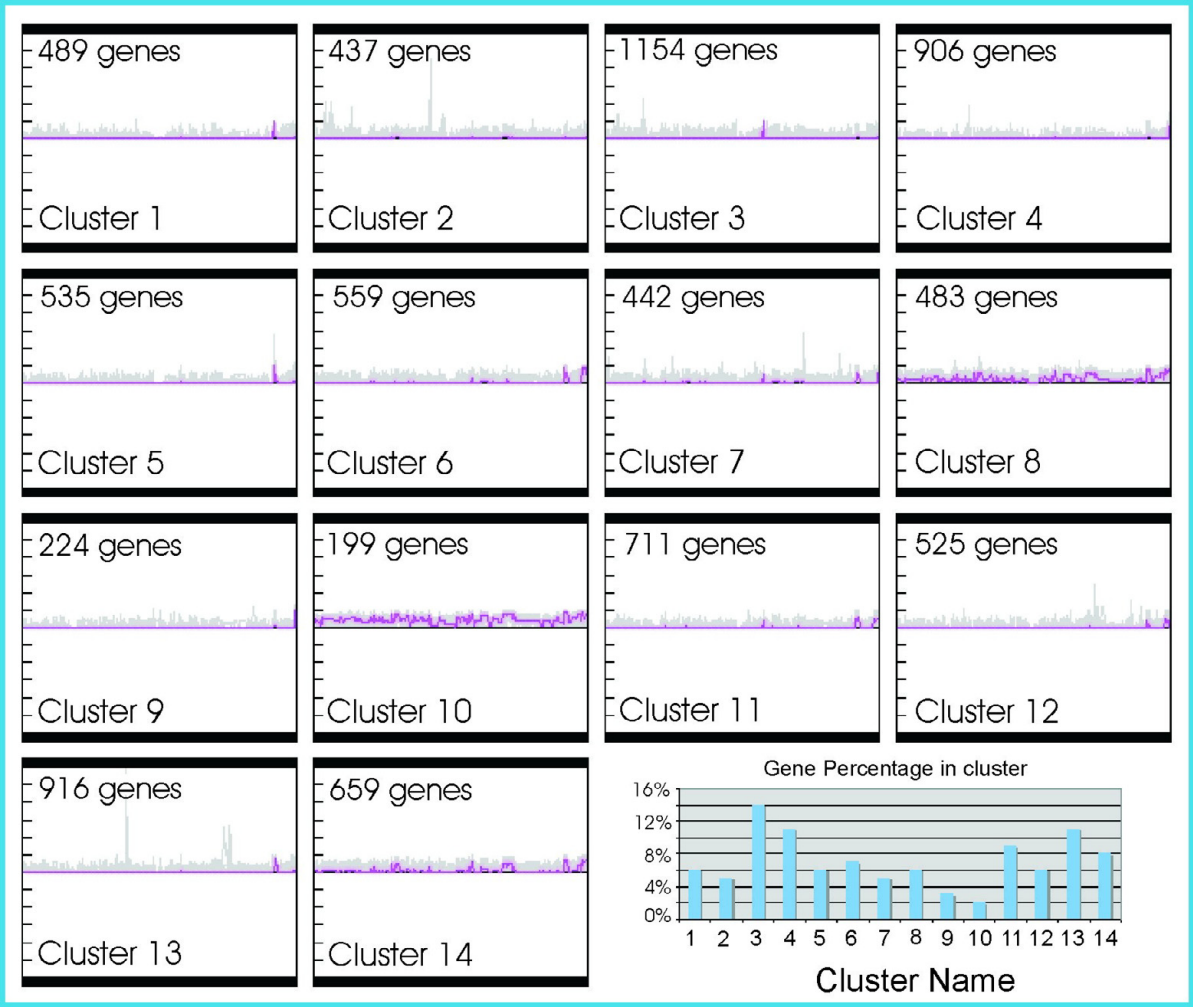
Phylogenetic profiles of all the 14 clusters identified by *k*-means analysis. Gray lines represent profile patterns (arrays of similarity measure) of genes in the clusters. Red line identifies cluster average profiles. Cluster name and number of genes belonging to clusters are reported into each box. The histogram on the right down box shows the percentage of genes belonging to each cluster.

We performed a *k*-means cluster analysis, setting the *k *value to 14. As shown in Fig. 4, the clusters 3, 4, 11, 13 and 14 contain the higher percentage of genes, accounting for more that 50% of the total genes, while clusters 9 and 10 contain the lower number of ORFs (3% of genes). The variance in each *k*-means cluster is very low (Fig. 4), meaning that the clusters contain genes with compact and similar profiles. As described in Fig. 4, the majority of the clusters (1, 2, 3, 4, 5, 6, 7, 9, 12, 13) contains genes with a similar profile, with the average values (red line) near zero, except for the presence of some spikes correspondent to an increasing similarity with some isolated organisms. As shown in Table 3, clusters 1, 3, 4, 5, and 9 contains genes that have a high similarity in a small subset of organisms. The majority of these ORFs are annotated as hypothetical proteins or phage related proteins. Clusters 8, 10 and 14 present genes shared among almost all the organisms. In particular cluster 10 is composed by the core genes described before having an high value of similarity widespread among all the organisms; cluster 8 contains genes shared mainly by gamma proteobacteria and cluster 14 is composed of genes in common between *Vibrionaceae *and *Enterobacteriaceae*.

A functional annotation has been performed on each gene cluster using COG (Cluster of Orthologous Genes), KEGG pathway map and GO databases. For each *k*-mean cluster the enrichment probability with respect to the total number of clusters has been obtained with the hypergeometric distribution.

**Figure 5:**
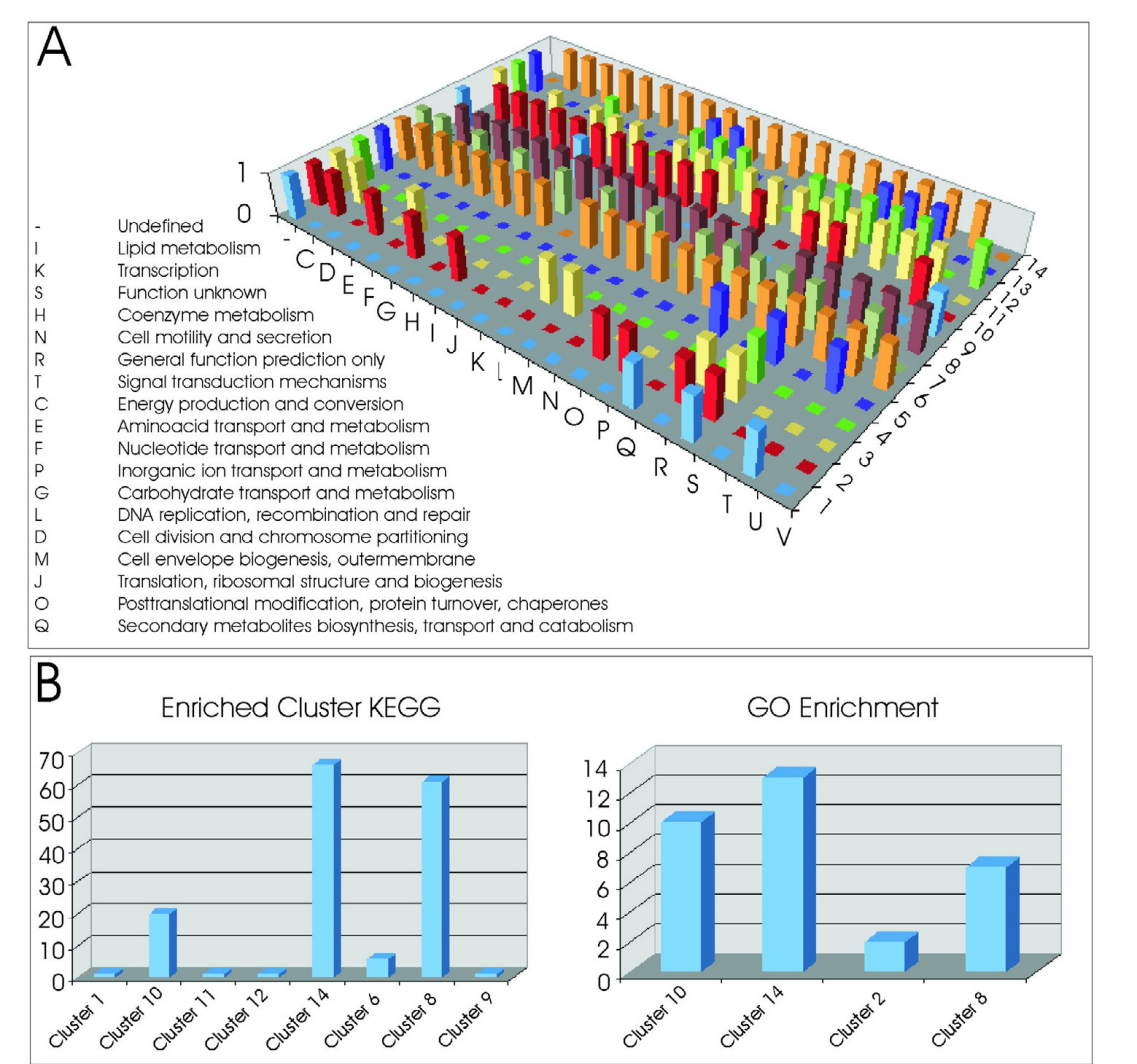
COG, KEGG and GO categories enrichment across the 14 *k*-means clusters. Panel A: Presence (coded 1) or absence (coded 0) of enriched COG categories for each cluster. Panel B: numbers of KEGG and GO enriched classes for each cluster.

Fig. 5 shows COG enrichment results for each cluster. As expected clusters represented by conserved genes (cluster 8, 10 and 14) have the higher number of enriched COG codes, while cluster specific of few organisms are characterized by a small number of enriched COGs. The majority of clusters presents COG codes enrichment for S (function unknown), R (poorly characterized) and – (absence of COG code) categories. This is due to the large abundance of unknown and hypothetical proteins presents in the *Vibrionaceae *proteomes.

It is worth noting that cluster 3, mainly represented by *Photobacterium profundum *SS9 ORFs, is enriched only by C (Energy production and conversion), L (DNA replication, recombination and repair) and M (Cell envelope biogenesis, outer membrane). Probably the L class overrepresentation is determined by the high number of transposons that are present in the SS9 genome [11]. The role played by these transposable elements in the survival of this deep-sea bacterium it is still a matter of debate [12].

In addition *V. vulnificus *YJ016 and *V. vulnificus *CMCP6 (cluster 13) seem to share genes belonging to the enriched COG classes K (Transcription), L and T (Signal transduction mechanisms). It was previously reported an enrichment in genes belonging to the transcription class in the genome of *V. vulnificus *respect to the *V. cholerae *genome [13]. This class is clearly related to the T class and seems to indicate that this organism is able to receive and translate in a transcriptional response specific environmental signals. Despite this, the large majority of the genes in clusters 3 and 13 lacks COG annotation.

Cluster 7, as shown in Table 3, accounts organisms with large genome size (see Table 1). This can explain the fact the this cluster contains almost all the COG class enriched and suggests a more complex and flexible life-style of these organisms compared to the other *Vibrionaceae *members.

KEGG annotation is limited to metabolic or structural complex network and so a reduced number of genes have a KEGG entry. This causes the presence of clusters without enriched map (cluster 2–5, 7, 13, see Fig 5). Also in this case, the clusters presenting the higher number of significant KEGG map are those containing the conserved genes. The most enriched KEGG clusters are cluster 14, 10 and 8 accounting for the majority of the metabolic pathways. Cluster 1 is enriched for map3080 (type IV secretion system). In fact *V. fischeri *genome contains 10 separate pilus gene clusters, including eight type-IV pilus loci. The presence of multiple pilus gene clusters suggests that different pili may be expressed in different environments or during multiple stages of its development as a symbiont [14].

Cluster 11 is enriched for map3090 (type II secretion system). The type II pathway is conserved among gram-negative bacteria, including many pathogens, and secretes a variety of virulence factors and degradative enzymes [15].

Cluster 9 is enriched for map 00860 (Porphyrin and chlorophyll metabolism). These genes are involved in the cobalamin (coenzyme B12) biosynthetic pathway [16]. Some organisms, such as *Salmonella typhimurium *and *Klebsiella pneumoniae*, can synthesize cobalamin *de novo *[17], while *E. coli *and large part of the *Vibrionaceae *perform cobalamin biosynthesis only when provided with cobinamide. It is interesting to observe that the genes belonging to the *de novo *pathway are only shared by *Archea*, some other organisms like *Salmonella*, *Pseudomonas *and *Vibrio *MED222.

Finally cluster 6 is enriched by map2010 (ABC transporter), map2020 (two-component system), map2030 and map2031 (bacterial chemotaxis), map2040 (Flagellar assembly) and map3090 (type II secretion system). This cluster contains genes shared with a high similarity by all *Vibrio *and with a lower similarity with *Photobacterium profundum *species. Among the *Vibrio *species the organisms showing the highest similarity (Tab. 3) are *V. cholerae *strains.

### *Vibrionaceae* specific genes

We identify 1940 clusters specific to the *Vibrionaceae*. All the *Vibrionaceae *considered in the analysis share 108 clusters. Among these genes we identify ToxR and ToxS genes. ToxR gene encodes a transmembrane regulatory protein firstly identified in *V. cholerae*, in which it co-ordinates many virulence factors in response to several environmental parameters [18]. *V. cholerae *ToxR activity is enhanced by a second transmembrane protein, ToxS, encoded downstream *tox*R [19]. This family of proteins is involved in response to temperature, pH, osmolarity and in *Photobacterium profundum *SS9, a piezophilic bacterium, to hydrostatic pressure [20]. The widespread presence of these genes among the *Vibrionaceae *suggests their importance in regulatory mechanisms.

**Figure 6:**
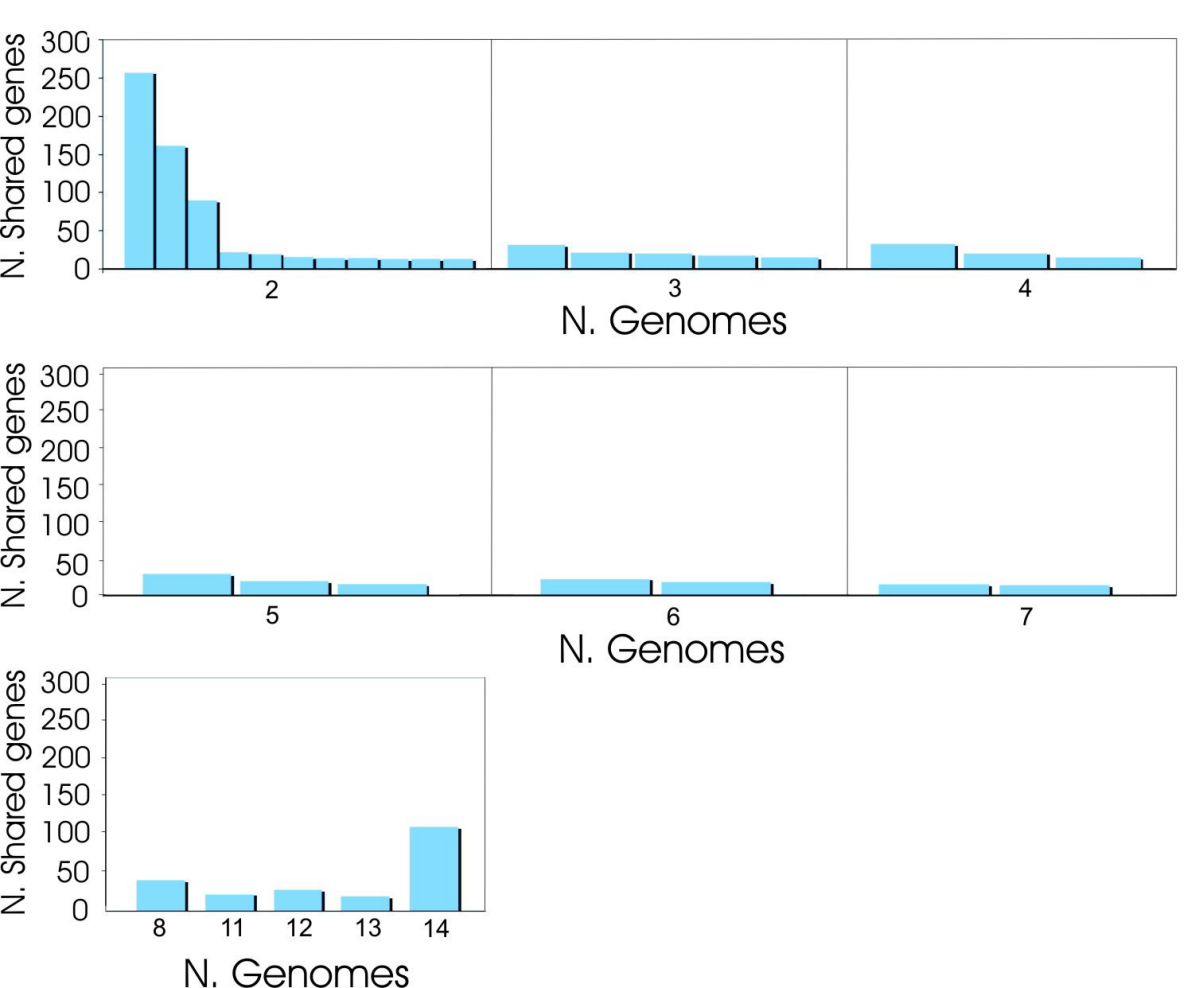
Number of gene clusters identified only in *Vibrionaceae *family; the number of *Vibrionaceae *genomes is reported on the x axis and the amount of shared genes is reported on the y axis. In the first histogram, for example, there are 11 groups of *Vibrios *each composed by 2 genomes.

We identify two other noteworthy groups of genes composed by 257 and 160 genes respectively shared just by two strains, mainly annotated as "hypothetical protein". The first group of genes is shared between *Photobacterium profundum *SS9 and *Photobacterium profundum *3TCK, while the second is shared between *V. vulnificus *CMCP6 and YJ016. These strains are closely related and this explains the high number of shared genes; while, inside the *Vibrionaceae *family, the number of specific shared genes highly decreases, showing a high inter-species variability (Fig. 6)

### Prophages and transposases

Prophages recover different biological roles both as quantitatively important genetic elements of the bacterial chromosome, and as vectors of lateral gene transfer among bacteria, due to their characters of mobile DNA elements. Indeed, numerous virulence factors from bacterial pathogens are phage encoded. It was postulated that this role of prophages is not limited to pathogenic bacteria but some adaptations of nonpathogenic strains to their ecological niche might also be mediated by prophages acquisition [21].

**Figure 7:**
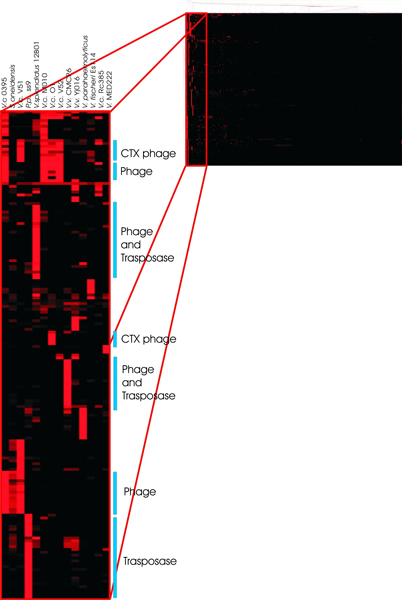
Two-way hierarchical cluster analysis performed on prophage and transposase proteins. The blue bars highlight the more interesting clusters of genes such as for example the *Vibrio cholerae *CTX prophage.

To better understand the importance of mobile elements within *Vibrionaceae *family, we performed a hierarchical cluster analysis using gene profiles annotated as "phage protein" and "transposase", for a total of 172 clusters of genes (Fig 7). We found that a high inter-strain genetic variability exists and phages and transposases are both shared by almost all *Vibrionaceae*, and specific to just some organisms. We identified five major clusters of mobile elements that are specific to a single organism. A group composed by 26 clusters containing both transposase and phage proteins seem to be unique to *V. splendiduds *12B01 (Fig 7). Another one composed by 16 clusters is specific of *V. vulnificus *CMCP6 (Fig 7) while *V. parahaemoliticus *has a cluster of 11 genes (Fig 7). Moreover there is another group of transposases and phage genes shared mainly by *V. cholerae *0395, *Shewanella oneidensis *and *V. cholerae *V51 (Fig 7). Finally a big cluster of almost 30 genes, all predicted to codify for transposases, was found in *P. profundum *SS9 genome (Fig. 7). The high presence of transposases in this bacterium seems to correlate with its deep-sea habitat, a feature presumably shared with other deep-sea microorganisms [12]. As shown in Fig 7, many of the clusters well conserved in an organism, are partially shared with a low similarity by other organisms. This agrees with the idea that prophages are not maintained in the genome over a long period of time and part of their genes may be deleted from the chromosome. Moreover, microarray analysis and PCR scanning demonstrated that prophages are frequently strain specific within a given bacterial species [22-24]. According to the modular theory of phage evolution, phage genomes are mosaics of modules, groups of genes functionally related, that are free to recombine in genetic exchanges between distinct phages infecting the same cell [21]. This can result in the occurrences of different part of phage distributed in far related genomes. Phylogenetic profile of some transposases is similar to the phage ones, suggesting a possible transfer mechanism phage-mediated for such mobile elements.

## Conclusion

In this work we propose an improved phylogenetic profile analysis on 14 *Vibrionaceae *genomes, to study this family on the basis of gene content. Using a phylogenetic profile for each cluster of genes defined as the median of all the profiles belonging to the cluster (meta-profile) we investigate the evolution of groups of ORFs belonging to the entire family. A two-way cluster analysis allows us to identify similarity structures on the entire phylogenetic matrix composed by 8,239 clusters of genes and 320 organisms.

The phylogenetic tree obtained with the cluster analysis does not reflect the global evolutionary tree because of the *Vibrionaceae *ORFs dataset used for the analysis, but rather can be considered as the *Vibrionaceae *perspective of bacterial diversity. The phylogenetic tree reflects the evolutionary processes that shape genomes, as lateral gene transfer, genes genesis and loss. In this context, the tree allows to group together genomes on the base of their global gene content.

We found that genomes belonging to the same taxonomic group tend to cluster together and that *Vibrionaceae *species are closely related. Moreover organisms belonging to the same or closely related taxa split into different subgroups, confirming the existence of a high variability among lineages, due to genetic and evolutionary process such as lateral gene transfer, concerted evolution and genes duplication.

On the other hand several groups of genes characterised by different homogeneous profiles have been identified. In particular we found, 1) a set of conserved genes (with a high similarity values across all organisms) that reflects the "minimal genome" composition defined in other previous works; 2) a set of genes mainly shared by *Vibrionaceae *and other *Gamma proteobacteria *and 3) genes specific to different sets of *Vibrionaceae*.

Finally a further analysis on prophage and transposase has confirmed the high inter-strain genetic variability even among closely related species.

The increasing number of genomes included in this type of analysis surely add new sorces of variability and noise, anyway we think that the use of meta-profiles can be useful for complexity reduction and data analysis to study global gene evolution.

## Methods

### Datasets

The *Vibrionaceae *species used in this analysis were selected among the freely available complete and draft genome sequences. The proteomes of *V. cholerae *N16961, *V. parahaemolyticus*, *V. vulnificus *YJ016, *V. vulnificus *CMCP6, *V. fischeri *ES114, *Photobacterium profundum *SS9 were downloaded from the NCBI ftp site [25]. Protein sequences of *Vibrio cholerae *MO10, *Vibrio cholerae *0395, *Vibrio cholerae *RC385, *Vibrio cholerae *V51, *Vibrio cholerae *V52 were downloaded from the NCBI genome database, while sequences of *Vibrio *MED222, *Vibrio splendidus *12B01, *Photobacterium profundum *3TCK from the J. Craig Venter Institute web site.

The 320 complete genomes update at 03/06 were downloaded from the NCBI ftp site.

### Similarity search and phylogenetic profile construction

All the *Vibrionaceae *ORFs were merged generating a redundant list of 59,669 proteins and were compared to all open reading frame from 320 bacterial and archeal genomes using Blastp. To determine the presence of an orthologous we used a combination of three different thresholds; a similarity value equal to or higher than 30%, an alignment length equal or higher than 70% and an Evalue score lower than or equal to e^-6^. After determining the presence of an orthologous gene, we computed a similarity index *I *for each pair of orthologous (a point of the phylogenetic profile) as follow:

I=SqsSqq⋅min⁡(lq,ls)max⁡(lq,ls)
 MathType@MTEF@5@5@+=feaafiart1ev1aaatCvAUfKttLearuWrP9MDH5MBPbIqV92AaeXatLxBI9gBaebbnrfifHhDYfgasaacH8akY=wiFfYdH8Gipec8Eeeu0xXdbba9frFj0=OqFfea0dXdd9vqai=hGuQ8kuc9pgc9s8qqaq=dirpe0xb9q8qiLsFr0=vr0=vr0dc8meaabaqaciaacaGaaeqabaqabeGadaaakeaacqWGjbqscqGH9aqpdaWcaaqaaiabdofatnaaBaaaleaacqWGXbqCcqWGZbWCaeqaaaGcbaGaem4uam1aaSbaaSqaaiabdghaXjabdghaXbqabaaaaOGaeyyXIC9aaSaaaeaacyGGTbqBcqGGPbqAcqGGUbGBcqGGOaakcqWGSbaBdaWgaaWcbaGaemyCaehabeaakiabcYcaSiabdYgaSnaaBaaaleaacqWGZbWCaeqaaOGaeiykaKcabaGagiyBa0MaeiyyaeMaeiiEaGNaeiikaGIaemiBaW2aaSbaaSqaaiabdghaXbqabaGccqGGSaalcqWGSbaBdaWgaaWcbaGaem4CamhabeaakiabcMcaPaaaaaa@532D@

where *l*_q _and *l*_s _are the query and subject length sequence respectively and *S*_qs _is the similarity score between the query and the subject sequence. S_qs _is defined as follow:

Sqs=∑i=1MαAqi,Asi+GP
 MathType@MTEF@5@5@+=feaafiart1ev1aaatCvAUfKttLearuWrP9MDH5MBPbIqV92AaeXatLxBI9gBaebbnrfifHhDYfgasaacH8akY=wiFfYdH8Gipec8Eeeu0xXdbba9frFj0=OqFfea0dXdd9vqai=hGuQ8kuc9pgc9s8qqaq=dirpe0xb9q8qiLsFr0=vr0=vr0dc8meaabaqaciaacaGaaeqabaqabeGadaaakeaacqWGtbWudaWgaaWcbaGaemyCaeNaem4Camhabeaakiabg2da9maaqahabaacciGae8xSde2aaSbaaSqaaiabdgeabjabdghaXjabdMgaPjabcYcaSiabdgeabjabdohaZjabdMgaPbqabaaabaGaemyAaKMaeyypa0JaeGymaedabaGaemyta0eaniabggHiLdGccqGHRaWkcqWGhbWrcqWGqbauaaa@4620@

where M is the match length between the query and subject sequence; Aq_i _and As_i _respectively the query and subject amino acid in position *i*; *α *the BLOSUM substitution matrix value for amino acid pair Aq_i_, As_i _and GP the gap penalty. GP is defined as follow:

GP = GOP+GEP(k-1)

where GOP is the Gap Open Penalty set to -11, GEP the Gap Extension Penalty set to -1 and k the gap length. *S*_*qq *_represents the score of the self-aligned query sequence.

S_qs _is always smaller than S_qq _and the score S range between 0 and 1. In order to take into account also the different sequence lengths, we multiplied the score S by the ratio between the minimum length between query and subject and the maximum length between query and subjects. In this way the total score is weighted on the base of the length, resulting in a lower similarity value if the lengths of the sequences are different.

The phylogenetic profile for each ORF is an array of index *I *with length equal to the number of genomes considered (320).

### ORFs clustering

The redundant list of 59,669 *Vibrionaceae *ORFs contained multiple copies of the same genes due to the presence of conserved genes in the considered genomes. In order to reduce the redundancy, we clustered proteins using a two-step approach. The first step is based on COG (Cluster of Othologous Genes) annotation. COG classifies conserved genes according to their homologous relationships. All the *Vibrionaceae *ORFs were annotated using COG clusters and proteins sharing the same COG code were considered belonging to the same cluster. In particular, the annotation process consists of a similarity search of all the ORFs against the COG proteins using blast and considering the best hit for each protein. 43,024 ORFs presented a similarity with a COG entry, producing 2,463 different clusters. In the second step, the remaining 16,645 ORFs without similarity with any COG entry were clustered using CD-HIT software [26]. CD-HIT program clusters protein sequence database at high sequence identity threshold and efficiently removes high sequence redundancy. This last clustering process produced 9,613 different groups of similar proteins.

Finally from the 12,076 total clusters obtained by this methodology, those composed by ORFs that do not have any ortologous genes (with a phylogenetic profile composed by an array with all zero values except for one position match with itself) were eliminated, resulting in a dataset composed by 8,239 distinct clusters.

The final phylogenetic profile for each cluster (meta-profile) was defined as the median of all the profiles belonging to the cluster. At the end of these procedures the final phylogenetic matrix was composed by 8,239 rows (cluster of genes) and 320 columns (organisms). In each cell the median of the index in the cluster was reported.

### Cluster analysis

Several clustering techniques have been used to identify the similarity structure underneath our data. A *k*-means and a two-way hierarchical cluster analysis with Euclidean distance and complete linkage were performed on the phylogenetic matrix.

The goal of a cluster analysis is to partition the elements into subsets without any constrains or *a priori *information, so that two criteria are satisfied: homogeneity, elements inside a cluster are highly similar to each other; and separation, elements from different clusters have low similarity to each other.

The Figure of Merit (FOM) is a measure of fit of the expression patterns for the clusters produced by a particular algorithm that estimates the predictive power of a clustering algorithm. It is computed by removing each sample in turn from the data set, clustering genes based on the remaining data, and calculating the fit of the withheld sample to the clustering pattern obtained from the other samples. On our data FOM analysis identified the best number of k-means clusters between 10 and 15. We decided to set *k *(in the *k*-means analysis) equal to 14. In each of these 14 clusters subsequent hierarchical cluster analysis was performed with bootstrap cluster assessment. All the previous analyses were performed with TMEV software [27], freely available at [28].

### Enrichment categories

Each cluster of genes has been annotated according to COG code, GO terms and KEGG pathway maps. Class enrichment (with respect to the entire matrix) has been calculated according to the hypergeometric distribution that was used to obtain the chance probability of observing the number of genes annotated with a particular COG, GO and KEGG category among the selected cluster. The probability P of observing at least *k *genes of a functional category within a group of *n *genes is given by:

P=∑i=kn(fi)(g−fn−i)(gn)
 MathType@MTEF@5@5@+=feaafiart1ev1aaatCvAUfKttLearuWrP9MDH5MBPbIqV92AaeXatLxBI9gBaebbnrfifHhDYfgasaacH8akY=wiFfYdH8Gipec8Eeeu0xXdbba9frFj0=OqFfea0dXdd9vqai=hGuQ8kuc9pgc9s8qqaq=dirpe0xb9q8qiLsFr0=vr0=vr0dc8meaabaqaciaacaGaaeqabaqabeGadaaakeaacqWGqbaucqGH9aqpdaaeWbqaamaalaaabaWaaeWaaeaafaqabeGabaaabaGaemOzaygabaGaemyAaKgaaaGaayjkaiaawMcaamaabmaabaqbaeqabiqaaaqaaiabdEgaNjabgkHiTiabdAgaMbqaaiabd6gaUjabgkHiTiabdMgaPbaaaiaawIcacaGLPaaaaeaadaqadaqaauaabeqaceaaaeaacqWGNbWzaeaacqWGUbGBaaaacaGLOaGaayzkaaaaaaWcbaGaemyAaKMaeyypa0Jaem4AaSgabaGaemOBa4ganiabggHiLdaaaa@47C6@

where *f *is the total number of genes with the same category (in the matrix) and *g *is the total number of genes in our matrix.

## Figures and Tables

**Table 1:** List of organisms used in the analysis.

**Organism Name**	**N. Genes**	**Life style**	**Status**
*Vibrio_cholerae*	3835	Pathogen	Complete
*Vibrio_fischeri_ES114*	3802	Symbiont	Complete
*Vibrio_parahaemolyticus*	4832	Pathogen	Complete
*Vibrio_vulnificus_CMCP6*	5024	Pathogen	Complete
*Vibrio_vulnificus_YJ016*	4488	Pathogen	Complete
*Photobacterium_profundum_SS9*	5491	Environmental	Complete
*Vibrio_cholerae_0395*	3505	Pathogen	Draft
*Vibrio_cholerae_MO10*	3418	Pathogen	Draft
*Vibrio_cholerae_RC385*	3221	Environmental ?	Draft
*Vibrio_cholerae_V51*	3323	Pathogen	Draft
*Vibrio_cholerae_V52*	3378	Pathogen	Draft
*Photobacterium_profundum_3TCK*	5531	Environmental	Draft
*Vibrio_MED222*	4590	Environmental	Draft
*Vibrio_splendidus12B01*	5231	Pathogen	Draft

**Table 2:** Core genes shared considering different number of genomes. The table shows the progressive number of genes shared with increasing number of genomes.

**N. of Genomes**	**Genes Annotation**
300	3-phosphoglycerate kinase
300	ABC-type phosphate transport system, ATPase component
300	Excinuclease ATPase subunit
301	Membrane GTPase LepA
301	Ribosomal protein S5
302	ABC-type dipeptide/oligopeptide/nickeltransport system, ATPase component
302	Aspartyl-tRNA synthetase
302	DNA-directed RNA polymerase, betasubunit/140 kD subunit
303	ABC-type oligopeptide transport system, ATPase component
303	Ribosomal protein S17
303	Ribosomal protein S3
303	ATPase components of various ABC-typetransport systems
303	Ribosomal protein S12
303	Ribosomal protein S7
304	Ribosomal protein S10
304	Ribosomal protein L1
305	CTP synthase UTP-ammonia lyase
305	ABC-type uncharacterized transportsystem, duplicated ATPase component
305	hydroxy methylpyrimidine transport ATP-binding protein
306	Glycine/serine hydroxymethyltransferase
306	Ribosomal protein S19
307	Signal recognition particle GTPase
307	Thioredoxin reductase
307	Enolase
307	ABC-type transport system involved inresistance to organic solvents, ATPase component
307	Signal recognition particle GTPase
308	Ribosomal protein S9
308	Seryl-tRNA synthetase
308	Ribosomal protein L2
308	ATP-binding protein
309	Cysteinyl-tRNA synthetase
309	ABC-type thiamine transport system, ATPase component
310	Metal-dependent proteases with possiblechaperone activity
310	Predicted ATPase involved in celldivision
310	Ribosomal protein L11
310	ABC-type arginine transport system, ATPase component
311	ABC-type polar amino acid transportsystem, ATPase component
311	ABC-type antimicrobial peptidetransport system, ATPase component
311	ABC-type histidine transport system, ATPase component
311	Ribosomal protein S11
311	Ribosomal protein L14
311	GTPases – translation elongationfactors
311	Predicted ABC-type transport systeminvolved in lysophospholipase L1 biosynthesis, ATPase component
312	Ribosomal protein L5

**Table 3:** Clusters of genes mainly shared by *Vibrionaceae *family. In the second column the organisms representative of each cluster (column 1) is reported with its median and standard deviation similarity profile.

**Cluster ID**	**Organism**	**Median value**	**Standard deviation**
**cluster 1**	*Vibrio fischeri ES114*	0.9	0.08
**cluster 2**	*Photobacterium profundum 3TCK*	0.95	0.13
**cluster 3**	*Photobacterium profundum SS9*	0.9	0.09
**cluster 4**	*Vibrio splendidus 12B01*	0.9	0.1
**cluster 5**	*Vibrio parahaemolyticus*	0.9	0.1
**cluster 6**	*V. cholerae*	0.8	0.15
	*V. vulnificus YJ016*	0.58	0.24
	*V. vulnificus CMCP6*	0.56	0.26
	*V. cholerae MO10*	0.88	0.12
	*V cholerae 0395*	0.87	0.14
	*V. cholerae RC385*	0.78	0.25
	*V. cholerae V51*	0.85	0.17
	*V. cholerae V52*	0.86	0.15
	*Vibrio MED222*	0.5	0.2
	*Vibrio splendidus 12B01*	0.51	0.28
**cluster 7**	*Photobacterium profundum SS9*	0.48	0.29
	*V. parahaemolitycus*	0.55	0.27
	*V. vulnificus CMCP6*	0.5	0.3
	*V. vulnificus YJ016*	0.54	0.28
	*Vibrio splendidus 12B01*	0.65	0.22
	*Photobacterium profundum 3TCK*	0.54	0.27
**cluster 9**	*Vibrio MED222*	0.97	0.12
**cluster 11**	*Photobacterium profundum SS9*	0.4	0.28
	*V. cholerae*	0.51	0.15
	*Vibrio fischeri ES114*	0.43	0.28
	*V. parahaemolitycus*	0.54	0.19
	*V. vulnificus CMCP6*	0.56	0.19
	*V. vulnificus YJ016*	0.57	0.19
	*V. cholerae MO10*	0.5	0.15
	*V cholerae 0395*	0.51	0.14
	*V. cholerae RC385*	0.36	0.22
	*V. cholerae V51*	0.42	0.2
	*V. cholerae V52*	0.48	0.17
	*Vibrio MED222*	0.52	0.22
	*Vibrio splendidus 12B01*	0.54	0.2
	*Photobacterium profundum 3TCK*	0.44	0.28
**cluster 12**	*V. cholerae*	0.41	0.44
	*V cholerae 0395*	0.5	0.45
	*V. cholerae MO10*	0.48	0.45
	*V. cholerae RC385*	0.28	0.4
	*V. cholerae V52*	0.35	0.43
**cluster 13**	*V. vulnificus CMCP6*	0.71	0.4
	*V. vulnificus YJ016*	0.77	0.35
